# Optimising Cannabidiol Delivery: Improving Water Solubility and Permeability Through Phospholipid Complexation

**DOI:** 10.3390/ijms26062647

**Published:** 2025-03-14

**Authors:** Thabata Muta, Riya Khetan, Yunmei Song, Sanjay Garg

**Affiliations:** Centre for Pharmaceutical Innovation (CPI), Clinical & Health Science, University of South Australia, Adelaide, SA 5000, Australiamay.song@unisa.edu.au (Y.S.)

**Keywords:** cannabidiol (CBD), controlled release, lipid-based nanotechnology, oral delivery, water solubility, phospholipid complexation (PLC)

## Abstract

Cannabidiol (CBD) has demonstrated therapeutic potential in treating epilepsy, multiple sclerosis, Alzheimer’s, Parkinson’s, and Crohn’s diseases. Despite its promising effects and analgesic, anti-inflammatory, and anxiolytic properties, oral CBD’s full potential is hindered by poor water solubility (0.7–10 μg/mL), low permeability, and chemical instability. This study aimed to enhance CBD’s dissolution, stability, and gastrointestinal (GI) permeability by forming a CBD–phospholipid complex (CBD-PLC). We hypothesised that CBD-PLC would enhance CBD’s hydrophilicity, thus improving GI barrier permeability. This study involved screening an optimal phospholipid (PL) using a Design of Experiments (DoE) approach to prepare CBD-PLC with nanosized droplets (194.3 nm). Dissolution studies revealed significantly enhanced release rates for CBD-PLC—44.7% at 2 h and 67.1% at 3 h—compared to 0% for pure CBD and 7.2% for a physical mixture (PM). Cellular uptake studies showed that at 30 µM, CBD-PLC exhibited 32.7% higher apparent permeability coefficients (P_app_), nearly doubling at 40 µM compared to pure CBD. Cytotoxicity tests confirmed safety over 24 h, while 12-month stability tests demonstrated consistent performance under varied conditions. The results indicate that CBD-PLC improves CBD’s solubility, permeability, and stability, offering a promising strategy to address the limitations of oral CBD delivery systems.

## 1. Introduction

Cannabidiol (CBD), a non-psychoactive cannabinoid derived from *Cannabis sativa*, has gained significant attention due to its potential therapeutic benefits in various health conditions, including antiseizure, analgesic, neuroprotective, anxiolytic, antidepressant, and antipsychotic effects [1]. Also, it has been shown to have antioxidative and anti-inflammatory properties [1,2]. CBD has a favourable safety and tolerability profile in humans [2]. For this reason, in 2018, the US FDA approved Epidiolex^®^, an oral solution containing CBD, for the treatment of seizures associated with Lennox–Gastaut syndrome, Dravet syndrome, or Tuberous Sclerosis Complex in patients one year of age and older [2,3]. This approval was supported by various clinical trials [3,4,5] and has led to rapid growth in CBD product consumption. Indeed, a report by Fortune Business Insights predicts that the global CBD market will grow from USD 7.59 billion in 2023 to an impressive USD 202.45 billion by 2032, with a compound annual growth rate of 43.66% from 2024 to 2032 [6].

Despite its potential, the application of CBD in clinical practice is limited by several challenges, including its poor water solubility (0.7–10 µg/mL) [7], physicochemical instability, and extensive first-pass metabolism [2,3], which are the main reasons for its low oral bioavailability (approximately 6% in humans) [8]. Moreover, the absorption of CBD shows high intra- and inter-subject variability, which can lead to inconsistent therapeutic outcomes [4]. For this reason, a significant number of formulation strategies have been explored, including the production of synthetic CBD [9], the development of self-emulsifying delivery systems [2,9,10], the encapsulation of CBD in gelatine matrix pellets [11], and the preparation of water-soluble CBD powders [12] and liposomes for the treatment of canine osteoarthritis pain [13].

Oil-based formulations are commonly used for cannabidiol (CBD) delivery; however, they present significant limitations. Despite their ability to improve solubility, they provide only a modest enhancement in bioavailability, with studies showing an increase of merely 14% compared to lipid-free formulations [14]. Additionally, CBD absorption from oil solutions is highly variable, as it is influenced by gastrointestinal conditions and food intake, leading to inconsistent therapeutic effects [14]. Moreover, CBD undergoes extensive first-pass metabolism, significantly reducing systemic availability [15]. Although many novel drug delivery systems have attempted to overcome CBD’s biopharmaceutical challenges, there is currently no lipid-based oral formulation containing CBD that has demonstrated a bioavailability improvement exceeding eight-fold [16], indicating the limitations of conventional lipid-based approaches. Furthermore, CBD in oil formulations degrades over time when exposed to heat, light, or oxygen, reducing its potency and shelf-life [15]. These drawbacks highlight the necessity for advanced delivery systems.

Phospholipid complexation (PLC) technology offers a promising drug delivery system for CBD, utilising phospholipids (PLs) to form lipid-compatible molecular aggregates. PLs, integral structural components of cell membranes, possess excellent biocompatibility, facilitating effective drug transport across biological barriers [17,18]. This delivery system not only provides controlled and targeted release but also enhances drug stability and shields gastrointestinal (GI) tissues from luminal enzymes [17,18]. Furthermore, PLC’s ability to enhance compound absorption reduces the necessary dosage of pharmaceuticals. PLs also contribute to nutritional and hepato-protective benefits, with complexes ranging in size from 50 nm to 100 μm, offering advantages in biodegradability and solubility, ultimately improving drug absorption and targeted delivery within the body [19,20]. The amphiphilic nature of PLs, particularly through their hydroxyl groups, allows for interaction with the polar components of hydrophobic compounds, forming a complex that enhances the drug’s therapeutic activity by improving its transport across lipid-rich biomembranes [18].

Indeed, previous studies have demonstrated that PLC significantly enhances the solubility and bioavailability of poorly water-soluble compounds. For instance, Quercetin exhibited a 12-fold increase in water solubility when formulated as a PLC [21]. Similarly, Silybin, a Biopharmaceutics Classification System (BCS) Class II drug—like cannabidiol (CBD) [22]—showed a remarkable 1265.9% improvement in relative bioavailability when formulated as a Silybin PLC [23]. These findings highlight the potential of PL-based formulations in overcoming solubility and absorption limitations, further supporting the rationale for employing this approach in CBD delivery.

Phosphatidylcholine (PC) is a PL that plays a critical role in maintaining cellular integrity and modulating various physiological processes. Its anti-inflammatory effects are well documented, with evidence showing that PC supplementation can prevent inflammatory responses and behavioural disturbances in disease models [24]. For instance, PC has been shown to ameliorate systemic inflammation and cognitive impairments by mediating the gut–brain axis balance, regulating neurotrophic factors, synaptic proteins, and gut barrier damage, thus improving gut health and reducing brain inflammatory responses [24]. To harness the potential of PC, this study investigates four distinct PLs, each with a PC content of at least 68%, to assess their efficacy in improving particle size, stability, and, most importantly, water solubility—a key objective of our research. Enhancing the water solubility of (BCS) Class II drugs, such as CBD [22], is crucial for increasing their bioavailability. The limited dissolution rate in the GI tract is the primary barrier to the absorption of these drugs [25]. BCS Class II drugs are characterised by high permeability but low solubility, leading to poor dissolution in the aqueous environment of the digestive system and consequently restricting their absorption into the bloodstream [25].

In line with these objectives, CBD-PLC was prepared using the solvent evaporation method. This complex was then characterised for its physicochemical properties using techniques such as Differential Scanning Calorimetry (DSC), Fourier Transform Infrared Spectrometry (FTIR), and Scanning Electron Microscopy (SEM), among others. Additionally, intestinal absorption was assessed using the Caco-2 monolayer assay to evaluate the permeability behaviour of CBD-PLC. A Quality by Design (QbD) approach was utilised to optimise the formulation, determining the effects of independent variables on dependent variables to select the most effective PL:CBD ratio and encapsulate the resulting CBD-PLC in acid-resistant gelatine capsules for oral delivery. Additionally, this study aimed to examine the in vitro release and permeation of the formulation and to comprehensively characterise the physical and chemical attributes and functional parameters of the fabricated complexes.

## 2. Results and Discussion

### 2.1. Quantification of CBD

The high-performance liquid chromatography (HPLC) method was used to determine the peak area of each standard and to generate the calibration curve. The mean regression equation for the concentration range of 5–100 µg/mL of CBD in methanol was run in triplicate, y = 80,926x – 10,018 (R^2^ = 0.9992, *n* = 7), where x represents the concentration and y represents the peak area of CBD.

The results from the liquid chromatography–mass spectrometry (LC-MS) method were obtained from eight concentration points ranging from 5 ng/mL to 1000 ng/mL, with six replicates (*n* = 6) for each point. The calibration curve followed the equation y = 101,766x + 398,014, demonstrating strong linearity with an R^2^ value of 0.9994. The limit of detection (LOD) was determined to be 1 ng/mL, while the limit of quantification (LOQ) was 5 ng/mL, and the retention time was 6.26 ± 0.03 min.

To ensure the reliability of the developed liquid chromatography–mass spectrometry (LC-MS) method, we assessed its intra-day accuracy and precision. The intra-day accuracy was evaluated by analysing multiple replicates of the samples under identical conditions within a single day, demonstrating consistent performance across measurements (Table 1). Additionally, the precision of the method was determined by assessing the repeatability and reproducibility of the obtained results, confirming the method’s robustness (Table 2). These findings validate the suitability of the LC-MS method for the accurate quantification of the target analytes.

### 2.2. Dynamic Light Scattering (DLS)

Initial measurements of the CBD-PLC formulation showed a particle size of 194.3 nm. Particles within the 50 nm to 200 nm range have been demonstrated to facilitate superior intestinal absorption, offering potential advantages for oral drug delivery systems. Conversely, particles exceeding 500 nm are associated with decreased transport efficiency across intestinal barriers, limiting their therapeutic utility [26].

Significantly, prior studies have achieved up to a 7.2-fold increase in oral bioavailability using particle sizes of around 329.63 ± 35.71 nm [27]. However, these results were dependent on complex procedures involving multiple solvents and extensive processing steps. In contrast, our streamlined methodology utilised ethanol, a safer solvent, achieving comparable particle size and enhanced polydispersity index (PDI) with greater stability and fewer processing requirements. Further research demonstrated that a drug–PL complex with a particle size near 7134.79 ± 0.67 nm improved solubility and pharmacological efficacy [28], underscoring the potential of the CBD-PLC formulation to similarly enhance bioavailability.

Additionally, the zeta potential of the formulation was measured at −32.5 mV, corroborating previous findings that a zeta potential above −30 mV is indicative of robust physical stability [29,30].

### 2.3. Lipophilicity Evaluation (N-Octanol/Water Partition Coefficient)

**Figure 1:**
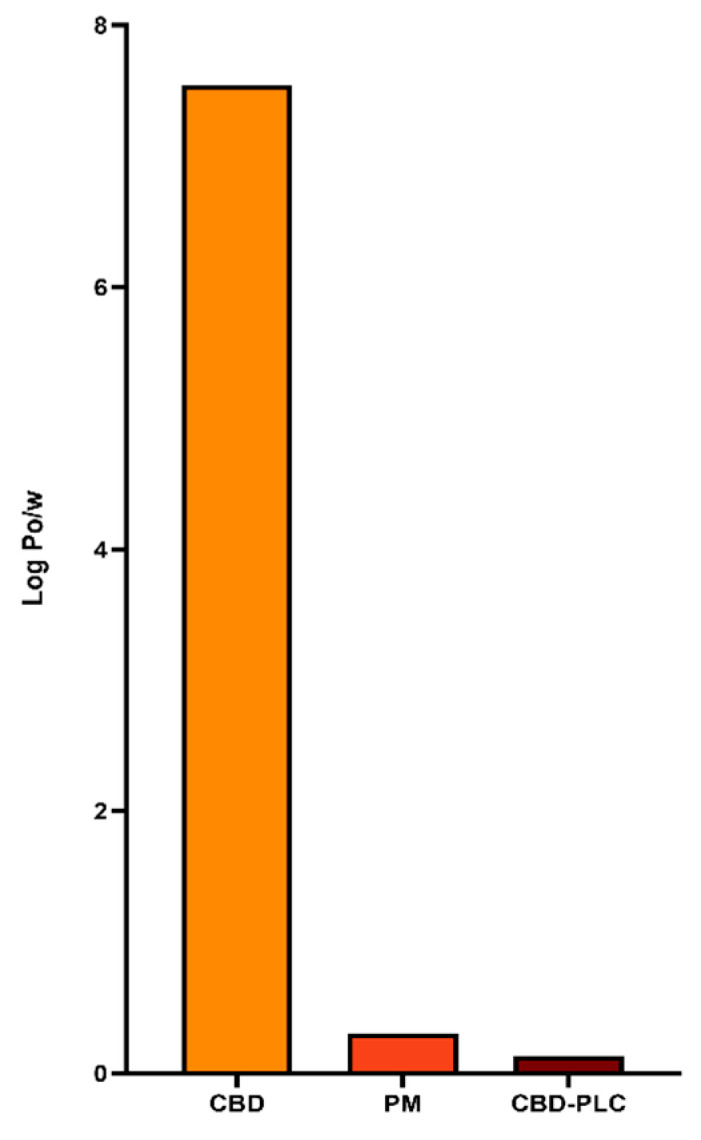
Logarithmic partition coefficients (log P_o/w_) for cannabidiol (CBD), physical mixture (PM), and cannabidiol–phospholipid complex (CBD-PLC) samples.

Figure 1 presents the logarithmic form of the oil/water partition coefficient (P_o/w_) profiles of CBD, a physical mixture (PM), and CBD-PLC. The data reveal that CBD has a notably high log P_o/w_ value of 7.54, highlighting its pronounced lipophilicity [31]. This is consistent with predictions from SwissADME which have shown a comparable log P_o/w_ value of 6.52 for CBD [32], further corroborating our experimental results. The high log P_o/w_ value suggests that CBD may experience limited bioavailability due to its poor aqueous solubility, which could hinder its absorption in biological systems, particularly in environments like the GI tract where water solubility is important [31]. In contrast, the PM of CBD and PL shows a significant reduction in the log P_o/w_ to 0.3. This decrease suggests that the physical presence of PLs in the mixture enhances the overall hydrophilic–lipophilic balance, likely due to the amphiphilic nature of the PLs, which can act as surfactants, increasing the solubility of lipophilic compounds like CBD by forming micellar structures or by dispersing them more effectively in aqueous environments [33,34].

**Figure 2:**
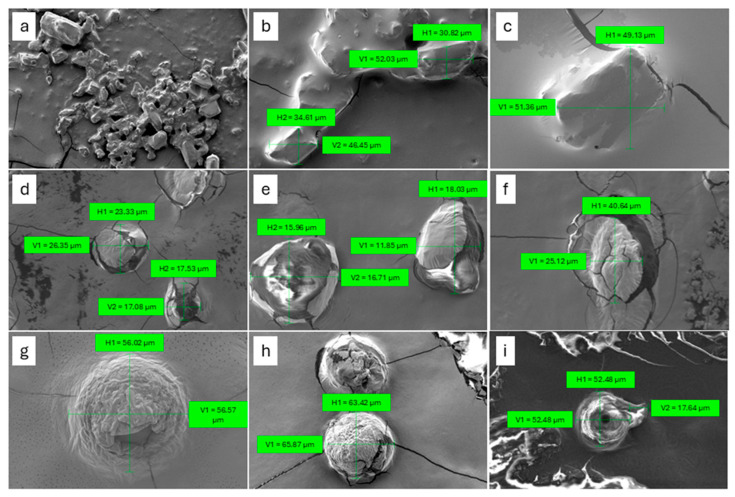
Scanning Electron Microscopy (SEM) images: (**a**) CBD at 100× magnification; (**b**) CBD at 500× magnification; (**c**) CBD at 1000× magnification; (**d**) PM showing CBD at 1000× magnification; (**e**) PM showing PL at 2530× magnification; (**f**) PM showing PL at 1000× magnification; (**g**) CBD-PLC at 1010× magnification; (**h**) CBD-PLC at 500× magnification; (**i**) CBD-PLC at 500× magnification.

Most notably, CBD-PLC exhibited an even lower log P_o/w_ value of 0.13, indicating a substantial reduction in lipophilicity compared to both CBD and the PM. This suggests that the complexation of CBD with PL has significantly altered the solubility profile of the compound. The formation of CBD-PLC likely improves CBD’s solubility in polar environments due to the encapsulating properties of the PL, which can facilitate the formation of a more stable, water-dispersible system [17]. Furthermore, PLCs, particularly those composed of highly saturated PLs, form nano-sized micelles, as demonstrated in the Scanning Electron Microscopy (SEM) images (Figure 2). These micelles significantly increase the equilibrium solubility of CBD-PLC in water [35]. This reduction in lipophilicity not only enhances solubility but could also improve CBD’s bioavailability by enabling better absorption and distribution within the body [17].

### 2.4. Preliminary Study for Selection of PL and Design of Experiments (DoE)

This study represents a significant advancement in the development of an oral CBD delivery system. The findings provide critical insights that will guide the optimisation of PL selection for further refinement using response surface methodology (RSM) within the Stat-ease 360 software (version: 23.1.3) framework. Notably, during the initial experimental phase, L-α-Phosphatidylcholine derived from soybean was excluded due to its suboptimal characteristics. It exhibited the largest particle size among the five PLs evaluated and showed the lowest solubility (log P_o/w_) in Phosphate-Buffered Saline (PBS) at pH 6.8, which is critical for effective CBD absorption in the small intestine [36]. These results underscore the importance of careful PL selection to enhance water solubility and potentially the bioavailability of oral CBD formulations.

The results from Table 3 and Table 4 indicate that although PL-100M exhibited the lowest Log P_o/w_, its particle size and PDI were not as favourable as those of PC-98T and L-α-Phosphatidylcholine derived from dried egg yolk. Particle size and PDI analysis demonstrated that the encapsulation of CBD with PC-98T resulted in particles with an average size of approximately half that of pure CBD (Table 4). Based on these findings, we employed the Design of Experiments to determine the optimal ratio (*w*/*w*) between the PL (PC-98T) and CBD for the most effective particle size, PDI and Log P*_o/w_* (Table 5).

The optimisation table (Table 6) demonstrates that our results fall within the range proposed by the DoE approach.

Among the PLs tested, PC-98T emerged as the most promising, showcasing superior attributes, as evidenced in Table 3 and Table 4. This led to its selection for subsequent optimisation using the DoE approach to determine the optimal CBD-to-PL ratio. The rigorous evaluation identified a specific ratio that accentuated the desired properties of CBD-PLC, as outlined in Table 6.

However, sourcing PC-98T posed a logistical challenge due to its limited availability in Australia. This necessitated exploring an alternative—L-α-Phosphatidylcholine from dried egg yolk. Preliminary observations supported this switch, showing that increasing the amount of any tested PL consistently amplified solubility (Table 3). This uniform behaviour underscores the foundational trait of PLs in the CBD formulation, transcending specific varieties.

Given this pervasive trend, it is plausible to infer that the optimised ratio obtained for PC-98T might be transferable to L-α-Phosphatidylcholine from dried egg yolk. While molecular interactions between PC-98T and L-α-Phosphatidylcholine from dried egg yolk may exhibit nuanced differences, their shared origin—egg yolk phosphatidylcholine—supports this hypothesis. By adopting the optimised ratio for L-α-Phosphatidylcholine from dried egg yolk, we leverage the intensive optimisation efforts previously undertaken, ensuring seamless research progression despite logistical challenges.

Furthermore, the established ratio between CBD and PL, 0.6783:20, as determined by the DoE, aligns with the maximum daily exposure (MDE) guidelines set forth by the US FDA. Notably, among all PLs analysed, L-α-Phosphatidylcholine from dried egg yolk delivered commendable secondary results in terms of particle size and PDI at pH 6.8 (Table 4), reinforcing its suitability as an alternative to PC-98T.

### 2.5. SEM

The PM of CBD and PL reveals that CBD forms crystals characterised by flat surfaces and sharp edges of irregular lengths (Figure 2a–c), while PL appears as rounded and irregularly shaped particles (Figure 2d–f). In contrast, CBD-PLC exhibits doughnut-shaped particles (Figure 2g–i), suggesting the probable dispersion of CBD within the PL matrix. The CBD-PLC particles display a notably smooth and porous topography with rounded edges, presenting a more ordered appearance and regular shapes (Figure 2g–i) compared to the individual CBD (Figure 2a–c) and PL (Figure 2d–f) particles [37,38].

Polymorphism is an essential part of pharmaceutical drug development since it can affect drug performance; for example, a difference of 4-fold in solubility can occur in different forms due to physicochemical properties and dissolution rate variances [39]. These dual or more different crystalline structures can potentially impact drug absorption and bioavailability [39]. Therefore, it is plausible to hypothesise that CBD-PLC particles may enhance solubility and dissolution rates.

### 2.6. Encapsulation Efficiency (EE)

The CBD-PLC and PL were found to be insoluble in acetonitrile, whereas pure CBD remained soluble in this solvent. This difference in solubility allowed for the quantification of pure CBD by measuring its concentration in the supernatant after centrifugation. All experiments were performed in triplicate to ensure accuracy and reproducibility. The EE% was calculated to be 78.53 ± 6.40%, demonstrating a highly efficient encapsulation of CBD within CBD-PLC.

### 2.7. Ultraviolet (UV) Spectra

**Figure 3:**
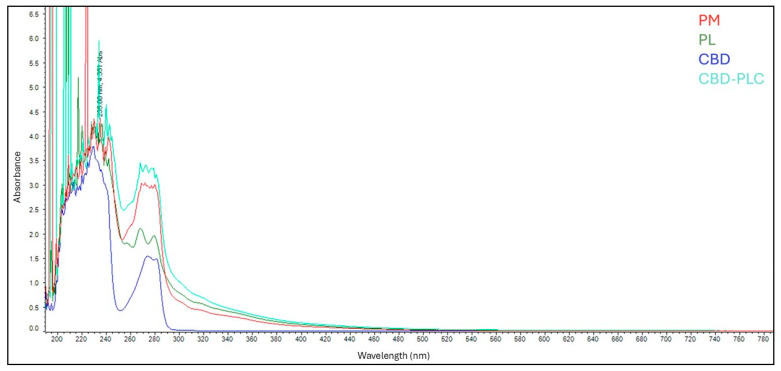
Ultraviolet (UV) spectroscopy results: red represents PM; dark green represents phospholipid (PL); blue is CBD; and light green is CBD-PLC.

The absorption curves of the PM (red) and CBD-PLC (light green) are almost indistinguishable (Figure 3). Conversely, the PL (dark green) and CBD (blue) displayed distinct absorption bands. This variation in the UV spectra of CBD upon interaction with the PL suggests potential chemical reactions between CBD and the PL’s functional groups [40].

### 2.8. Differential Scanning Calorimetry (DSC)

**Figure 4:**
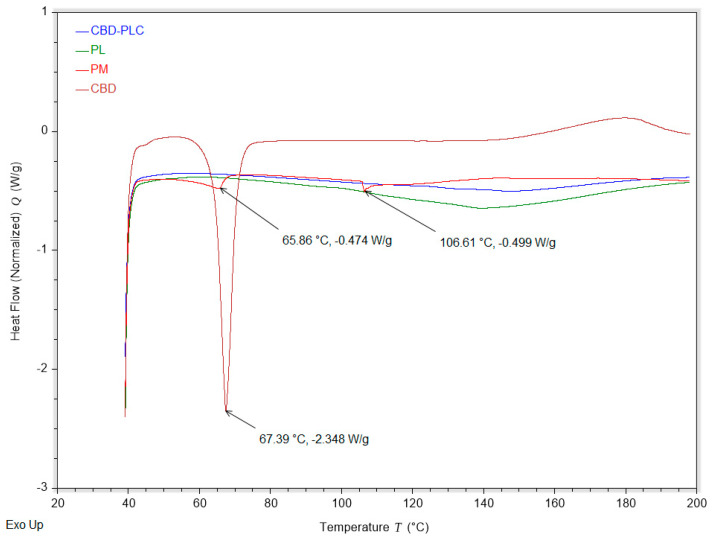
Differential Scanning Calorimetry (DSC) thermograms of cannabidiol (CBD, red), the phospholipid (PL, green), the physical mixture (PM, bright red), and the CBD–phospholipid complex (CBD-PLC, blue). The thermograms illustrate the thermal transitions of each component, with notable endothermic peaks observed at 67.39 °C (−2.348 W/g), 65.86 °C (−0.474 W/g), and 106.61 °C (−0.499 W/g). The data provide insights into the thermal behaviour, crystallinity, and interaction between CBD and phospholipids within the complex. Exothermic transitions are oriented upwards.

The DSC analysis of CBD, the PL, the PM, and the complex (CBD-PLC) revealed distinct thermal behaviours for each sample (Figure 4). The CBD showed a sharp endothermic peak at around 66 °C, which is consistent with its melting point, as reported in the literature [13]. The PM of CBD and the PL exhibited the CBD peak, suggesting that the two components retain their individual characteristics in the mixture. However, the complex showed a significant change in the DSC curve, with the disappearance of the CBD peak. This could be indicative of the incorporation of CBD into the PL structure, possibly due to the formation of the complex. This observation aligns with the findings of Shilo-Benjamini, Y. et al. [41], who reported the successful encapsulation of CBD. This could have significant implications for the development of CBD formulations, which have been shown to enhance the bioavailability and therapeutic efficacy of CBD [13,41].

### 2.9. Fourier Transform Infrared Spectrometry (FTIR)

**Figure 5:**
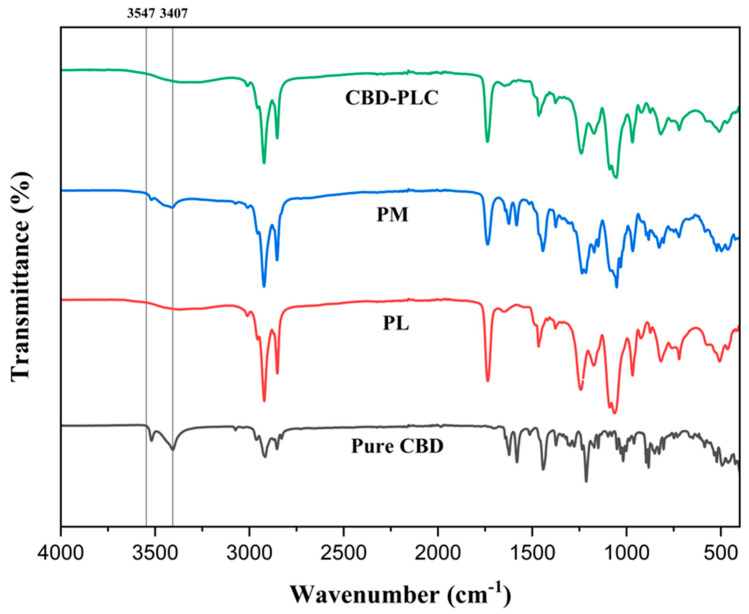
Fourier Transform Infrared (FTIR) Spectroscopy results: CBD-PLC (green); PM (blue); PL (red); and CBD (black).

FTIR offers a profound understanding of molecular interactions between active pharmaceutical ingredients and excipients. The spectra of CBD, the PM (comprising CBD and the PL at the optimised ratio of 0.6783:20), and CBD-PLC were studied to discern potential interactions between CBD and the PL (Figure 5). The FTIR spectrum of CBD (grey) reveals significant molecular vibrations within the range of 3407 to 3547 cm^−1^ which correspond to the O-H (aromatic) stretching vibrations [42]. While these molecular vibrations are evident within the range of the pure drug (79% T) and the PM (92%T), a shift to a diminished peak at 3379 cm^−1^ in the complex is observed, aligning with the peak at 3376 cm^−1^ exclusive to the PL. Zhang et al. (2014) suggested that several signals in the initial segment of the FTIR spectrum arise from O-H and N-H stretching vibrations [43]. Their disappearance in the complex, compared to the PM, potentially indicates the formation of hydrogen bonds between CBD and PC [43]. This suggests that the hydroxyl (O-H) or amine (N-H) groups present in the PM might be engaged in hydrogen bonding within the complex, leading to the disappearance or shift of these peaks [43]. Furthermore, the bands proximate to 3000 cm^−1^ are ascribed to C-H stretching (phenyl) [42], and the sharp peak around 2920 cm^−1^ indicates the presence of methyl and methylene groups [42]. In addition, the C-O stretching vibrations are detected at approximately 1215 cm^−1^ [42].

**Figure 6:**
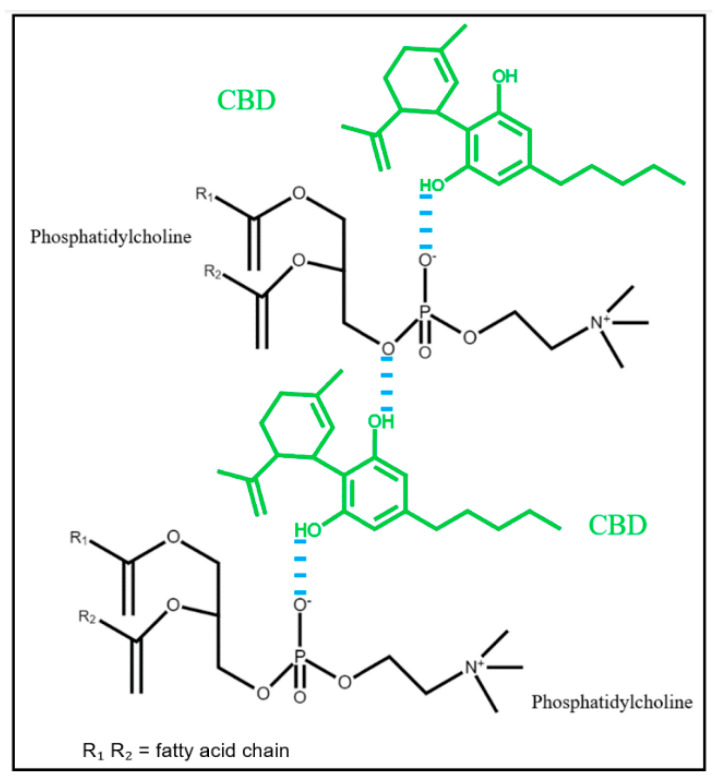
Proposed interaction mechanism between CBD and PLs.

The FTIR spectra of CBD-PLC predominantly reflect the peaks of CBD and the PM. For the pure drug and PM, the region ~1580 cm^−1^ illustrates C=C stretching (phenyl ring) [42], with benzene ring stretching evident at 1646 cm^−1^ [27]. The absence of these peaks in the formulation and their nonexistence in the PL suggest a modification in the molecular environment surrounding the aromatic rings of CBD upon complexation with PC [42]. Such alterations might be indicative of the formation of CBD-PLC through weak physical interactions and could be attributed to π-π stacking interactions, van der Waals forces, or other non-covalent interactions influencing the vibrational frequencies of these bonds, such as hydrogen bonding or van der Waals interactions between the –OH group of CBD’s phenyl rings and the polar end (P=O group) of the PLs, as proposed in Figure 6.

The peaks observed in Figure 5 at 2921 cm^−1^, 2852 cm^−1^, and 1738 cm^−1^ could be assigned as characteristic C-H stretching bands of long-chain fatty acid and carbonyl (C=O) stretching bands of a PL, respectively [27]. The absorption profiles of these PL peaks remain unchanged in the FTIR spectra of CBD-PLC (Figure 5) and the PM (Figure 5). This observation highlights that the non-polar saturation of the long-chain fatty acids intrinsic to the PL did not participate in the formation of the complex [27]. Moreover, the FTIR analysis confirms that the structure of CBD remains unaltered post-complexation. However, peak shifts potentially due to hydrophobic interactions are evident, culminating in the formation of CBD-PLC [27].

#### Principal Components Analysis (PCA) for FTIR

**Figure 7:**
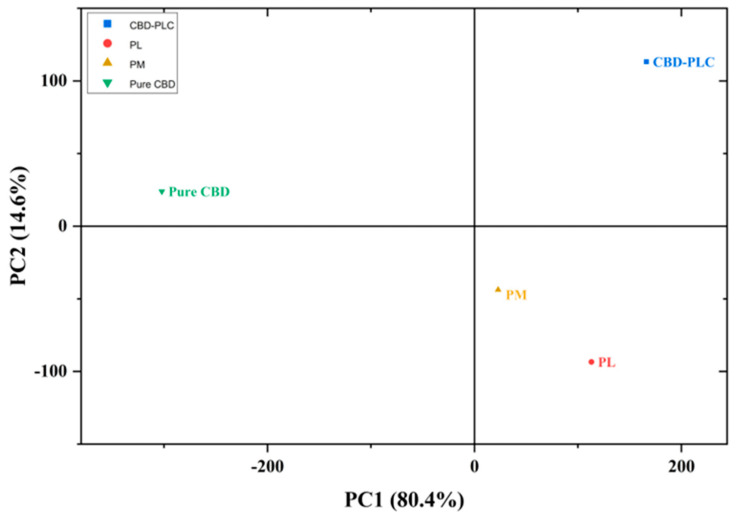
Observation plot for PCA.

To further explore the variance within the FTIR spectra, PCA was performed and enabled the identification of key features contributing to the differentiation among samples (Figure 7). Along PC1 (80.4%), which captures the majority of variance, pure CBD loads negatively, while the PM, the PL, and CBD-PLC load positively. The positioning along PC1 appears to be primarily influenced by compositional differences among the samples, indicating their distinct physicochemical properties [44]. The loading of the PM in the positive region of PC1 suggests that while the PM retains characteristics of both components, its spectral and structural properties are still distinct from those of pure CBD [44]. On the other hand, CBD-PLC and the PL are positioned closer to each other along PC1, indicating that the high PL content (>95%) in CBD-PLC is the dominant factor influencing its properties, leading to strong spectral and physicochemical similarities [45].

PC2 (14.6%) accounts for a smaller proportion of variance but provides critical insights into structural transformations. Pure CBD and CBD-PLC load positively on PC2, whereas the PL and PM are positioned negatively. The positive loading of pure CBD along PC2 suggests that it retains its highly ordered crystalline structure, a characteristic confirmed by DSC, which showed a sharp endothermic melting peak (Figure 4). The strong positive loading of CBD-PLC along PC2, despite its high PL content, suggests that CBD-PLC exhibits distinct structural characteristics compared to the PL [46]. This observation supports the hypothesis that interactions between CBD and the PL occurred during the formulation process, possibly leading to molecular dispersion and reduced crystallinity [45], as indicated by the absence of a defined melting peak in DSC and hydrogen bonding interactions observed in FTIR (Figure 5).

Pure CBD and CBD-PLC are well separated from all other samples, confirming their distinct nature [46]. The proximity of the PM to the PL suggests a similarity in properties between the samples [45]. Overall, the separate positioning of the PM to CBD-PLC confirms that the physical mixing alone does not lead to significant molecular interactions [46], reinforcing that the formulation process has led to molecular interactions and structural modifications [44,45].

### 2.10. ^1^HNMR

**Figure 8:**
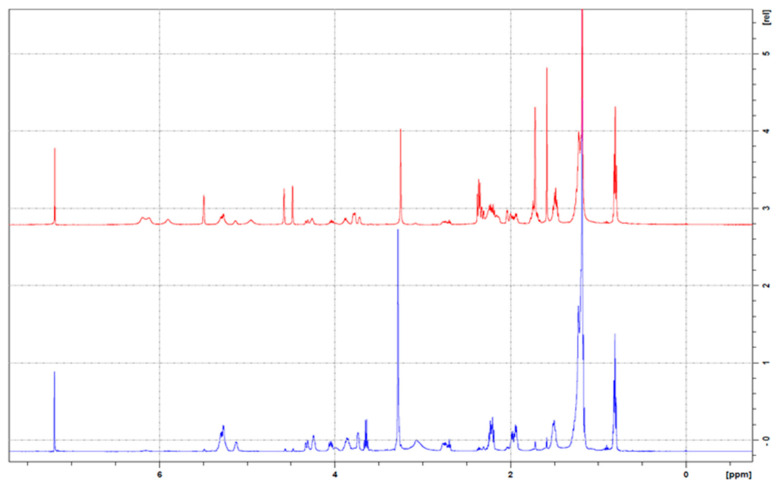
^1^H nuclear magnetic resonance (NMR) spectra: overlay of PM (red) and CBD-PLC (blue).

**Figure 9:**
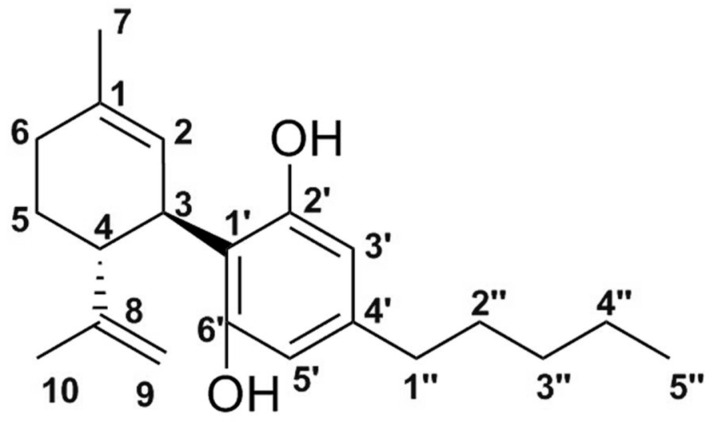
Chemical structure of CBD [52].

To investigate the structural attributes of the inclusion complexes, the ^1^H NMR spectra of CBD, the PL, the PM, and the resultant inclusion complexes were analysed. The ^1^H NMR spectrum of CBD (Figure 8) aligns with the established spectral characteristics documented in the literature [47,48,49,50,51]. Pertaining to the complex, notable observations include the spectral behaviour of protons H-3′ and H-5′ and hydroxyl groups OH-2′ and H-2 (Figure 9). The peaks characteristic of the pure drug spectra are conspicuously absent in the spectrum of the complex. This observation lends substantial support to our proposed interaction between the PL and CBD, suggesting that the interaction indeed alters the spectral signature in a manner consistent with our hypothesis.

In the context of the PL, it is evident that chemical shifts play a pivotal role in NMR spectroscopy, serving as indicators of the electronic environment surrounding nuclear spins. Shifts in peak positions, particularly within the complex, are indicative of alterations in this electronic environment, potentially due to molecular interactions [53]. Furthermore, variations in peak intensities are observed, which are often reflective of changes in the relaxation properties of nuclei. These variations can be attributed to molecular interactions [54].

Observational Data:

1. PL: Peaks at 4.4025 ppm (intensity: 6.16 × 10^−1^), 4.1574 ppm (1.80 × 10^−1^), 3.9972 ppm (4.60 × 10^−1^), and 3.9123 ppm (4.82 × 10^−1^).
2. PM: Peaks at 4.4587 ppm (1.99 × 10^−1^), 4.3388 ppm (9.82 × 10^−2^), 4.2625 ppm (4.33 × 10^−1^), 4.0880 ppm (1.64 × 10^−1^), and 3.9136 ppm (1.99 × 10^−1^).
3. CBD-PLC: Peaks at 4.4805 ppm (5.02 × 10^−2^), 4.4369 ppm (4.23 × 10^−2^), 4.3497 ppm (1.49 × 10^−1^), 4.2516 ppm (4.84 × 10^−1^), and 4.0662 ppm (2.56 × 10^−1^).
4. CBD: Peaks at 4.5940 ppm (1.85) and 3.9095 ppm (4.79 × 10^−1^).

### 2.11. In Vitro Drug Dissolution Study

**Figure 10:**
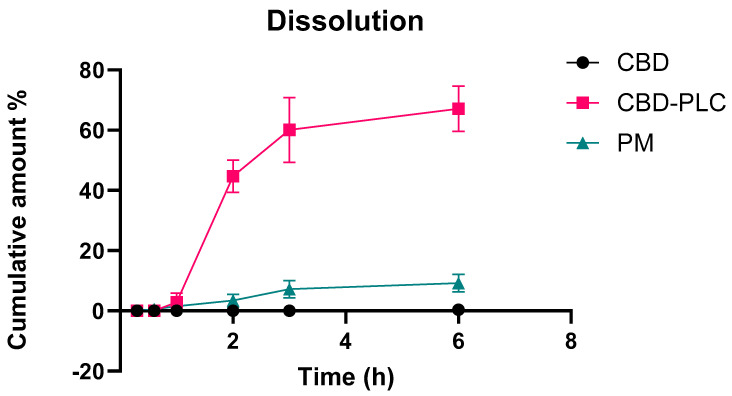
The dissolution profiles of three samples—pure CBD (black curve), CBD-PLC (red curve), and a PM (green curve)—are presented, with all samples encapsulated in acid-resistant capsules. Cumulative drug release is plotted against time to depict both the rate and extent of CBD dissolution. Each time point represents the mean cumulative release (±SD) based on triplicate measurements (*n* = 3). This analysis provides comparative insights into the dissolution behaviour of the different formulations over the observed period.

Figure 10 demonstrates a release rate of 44.7% at 2 h and 67.1% at 3 h, highlighting the efficacy of PLs as a delivery mechanism compared to the 0% release rate for pure CBD and 3.5% and 7.2% for the PM at the same time points. These results align with the previous literature. Zhenhai Zhang et al. reported a release rate of approximately 20% after 2 h [55], while Yingpeng Tong et al. described a similar release profile, characterised by an initial rapid dissolution (~50%) within the first 0–2 h, followed by a more gradual increase between 2 and 12 h, culminating in near-complete dissolution of the PLC by 12 h [40]. Notably, their data indicated a 50% release at 2 h, progressing to around 60% by 6 h, a trend that closely mirrors our observations. In studies utilising comparable methodologies, dissolution rates of 35–50% at the 2 h mark were commonly observed.

Previous dissolution outcomes did not align with the anticipated profile, which expected a rapid release within the first two hours, followed by a gradual increase extending to the 12 h mark [49]. Upon recognising that the dissolution medium might be a limiting factor, solubility studies of CBD-PLC were undertaken (Table 7). Various dissolution media, designed to simulate biological environments, were assessed to identify the most suitable medium for in vitro drug dissolution studies. CBD-PLC exhibited poor stability in PBS, whether prepared with Reverse Osmosis (RO) water or MilliQ water, likely due to ionic interactions that could interfere with the complex and produce inaccurate results. The presence of ions in PBS can interfere with lipid dissolution tests due to specific ionic interactions with PL components. For example, the study by Brinkmann-Trettenes and Bauer-Brandl (2014) found that the dissolution rate of griseofulvin in solid PL nanoparticles was significantly different when tested in PBS compared to other media, indicating that the ionic content of PBS can alter the dissolution properties of lipid-based systems [56]. Similarly, Salehi et al. (2020) highlighted that the dissolution of ionizable drugs was slower in phosphate buffer compared to bicarbonate buffer, due to differences in buffer capacity at the interfacial layer [57]. This suggests that the phosphate ions in PBS could similarly affect the stability and dissolution of phospholipid-based formulations [57]. Due to the significantly enhanced recovery of CBD in MilliQ water, as demonstrated in Table 7, this medium was selected for subsequent dissolution studies, aligning with the best practices outlined in USP guidelines [58].

**Figure 11:**
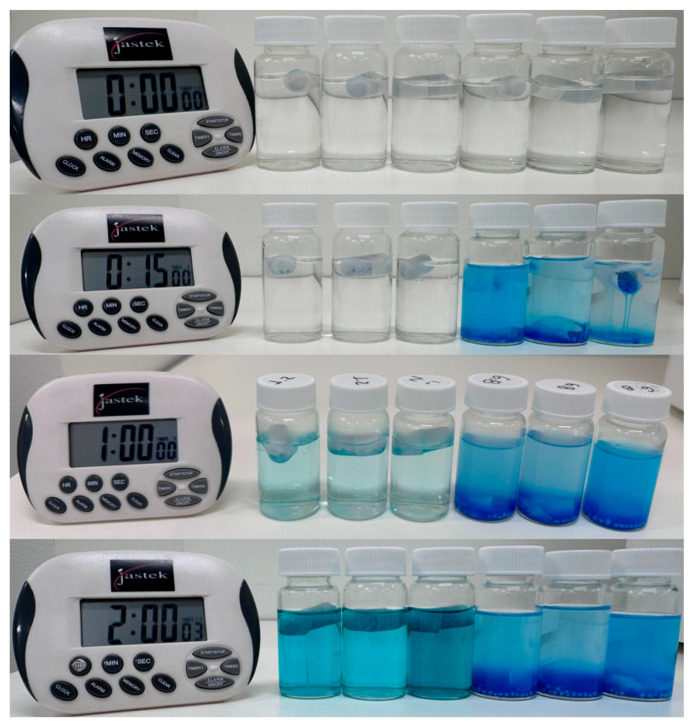
Time-dependent assessment of capsule integrity in different media. The three flasks on the left contain capsules in a pH 1.2 solution, and the three flasks on the right contain capsules in MilliQ water (pH 5.1). The image captures the state of the capsules at 0 h, 15 min, 1 h, and 2 h, highlighting the delayed disintegration in acidic conditions compared to the rapid release at pH 5.1.

As part of our preliminary studies, we conducted an integrity evaluation of the selected capsule in media with different pH values. The capsules were filled with blue food colouring pellets and the blue colour was used as a capsule release indicator (Figure 11). The capsules were placed in a pH 1.2 solution and MilliQ water (pH 5.1) and incubated at 37 °C under constant agitation for 2 h to evaluate their integrity.

**Figure 12:**
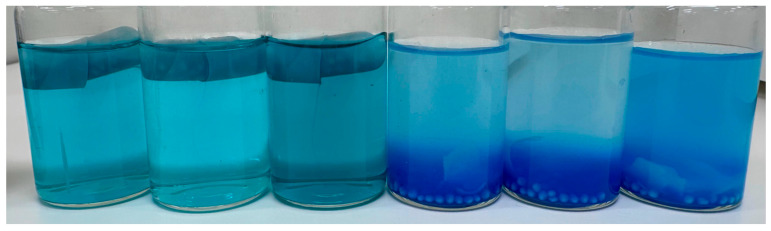
Capsule integrity after 2 h in different media. The three flasks on the left contain capsules in a pH 1.2 solution, where they remained intact with no pellet release. The three flasks on the right contain capsules in MilliQ water (pH 5.1), where they fully disintegrated, releasing the pellets, visible at the bottom.

In MilliQ water (pH 5.1), complete pellet release occurred within 15 min, indicating rapid capsule disintegration. In contrast, in the acidic medium (pH 1.2), pellet release was significantly delayed, with only a tiny amount of colour diffusion observed after 1 h. By the 2 h mark, partial dissolution of the release medium had led to some internal degradation of the pellets, as evidenced by the blue coloration; however, the capsules remained structurally intact (Figure 12).

Given that the pH of the MilliQ water used in the experiments fluctuated between 5 and 6, additional release studies at pH 4.5 and 6.8 were deemed unnecessary. Similarly, dissolution studies at pH 1.2 were not conducted, as the capsules can retain their integrity for at least 2 h in this acidic condition.

### 2.12. Stability Study

CBD-PLC’s initial concentration was established. CBD-PLC remained to be stable after 12 months at 4 °C and 25 °C. The values in Table 8 fall well within the stability range set by both the FDA and TGA, indicating good stability at these temperatures.

However, at 40 °C, CBD-PLC retained only 86.75 of its initial concentration, which is below the stability threshold set by the FDA (90–110%) [59] and TGA (92.5–107.5%) [60]. This reduced stability at 40 °C is further corroborated by a noticeable change in colour to a darker hue. This physical change, along with the decrease in concentration, suggests that CBD-PLC demonstrates less stability at higher temperatures, particularly at 40 °C, over a 6-month period. Despite this decrease in concentration, it is noteworthy that there were no significant changes in other characteristics such as PDI and zeta potential. An example particle size and zeta potential distribution of CBD-PLC on day 0 is shown in Supplementary Figures S1 and S2, respectively.

### 2.13. Intestinal Cell Monolayer Preparation and Treatment

#### 2.13.1. Assessment of Cell Viability in Caco-2 Cells

**Figure 13:**
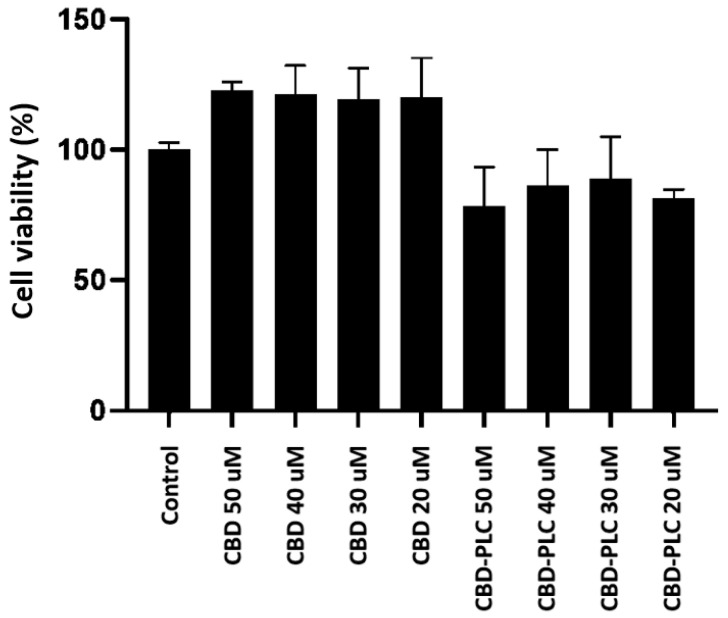
The MTT assay results indicating cell viability, *n* = 3 ± SD. The x-axis and y-axis represent samples and cell viability (%), respectively.

The consolidated data from the MTT assay over a 24 h period demonstrate that CBD-PLC, at concentrations of 20 µM, 30 µM, and 40 µM, does not exhibit significant cytotoxic effects on the tested cell line (Figure 13). The average cell viability percentages for these concentrations are 81.39%, 88.72%, and 86.33%, respectively, all of which are above the 80% threshold commonly used to indicate nontoxicity [61]. Even at the highest tested concentration of 50 µM, the average cell viability is 78.21%, which is still aligned with the toxicity threshold, suggesting that CBD-PLC is likely nontoxic at this concentration as well, especially for short-term exposure as expected in oral formulations.

This observation is particularly relevant for oral formulations of CBD-PLC, which are not anticipated to remain in the human body for more than 24 h [62]. The absence of cytotoxicity within this timeframe aligns with the expected duration of exposure in vivo, indicating a favourable safety profile for short-term use. Additionally, these results are instrumental in identifying appropriate concentrations and time points for permeation studies, essential for assessing the permeation of CBD-PLC.

#### 2.13.2. Cell Viability and Transepithelial Electrical Resistance (TEER) Assay

**Figure 14:**
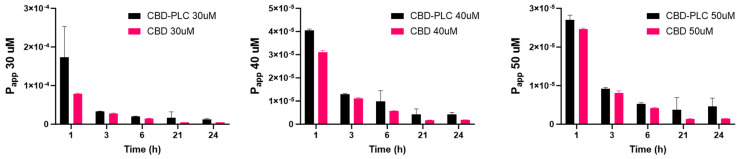
P_app_ (cm/s) of CBD and CBD-PLC at 30 µM, 40 µM, and 50 µM over 24 h, with *n* = 3 ± SD.

The permeability data for CBD and CBD-PLC at three different concentrations (30 µM, 40 µM, and 50 µM) were evaluated over a 24 h period (Figure 14). The apparent permeability coefficient (P_app_) values clearly indicate superior performance of the CBD-PLC formulations across all concentrations when compared to pure CBD.

**Figure 15:**
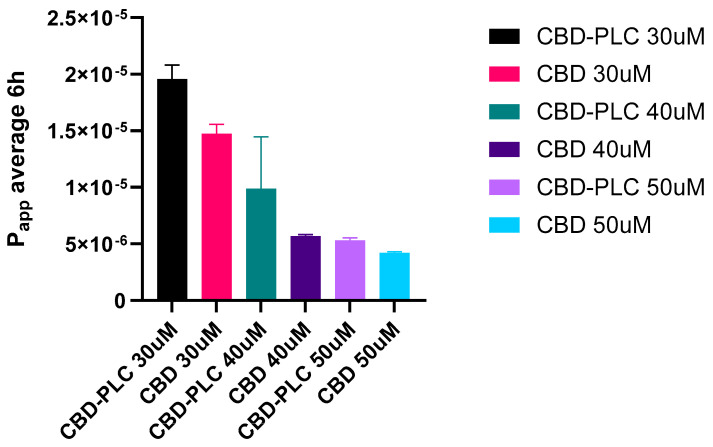
P_app_ (cm/s) of CBD transport from apical to basolateral side, with *n* = 3 ± SD.

At the 30 µM concentration (6 h), the P_app_ of CBD-PLC (1.96 × 10^−5^ cm/s) was significantly higher than that of pure CBD (1.48 × 10^−5^ cm/s), reflecting a 32.7% improvement in permeability (Figure 15). A similar trend was observed at 40 µM, where the CBD-PLC formulation (9.87 × 10^−6^ cm/s) displayed nearly double the permeability of pure CBD (5.71 × 10^−6^ cm/s). At 50 µM, CBD-PLC (5.32 × 10^−6^ cm/s) maintained superior permeability compared to pure CBD (4.22 × 10^−6^ cm/s), showing a 26.1% increase in P_app_.

The recovery percentage (6 h) in Table 9, which indicates the amount of drug recovered from the basolateral side after transport, is significantly higher for CBD-PLC. At 30 µM, the recovery for the complex is 7.5%, compared to only 1.2% for CBD. This difference becomes more pronounced at higher concentrations, with recoveries of 22.8% vs. 3.03% at 40 µM and 35.3% vs. 5.18% at 50 µM.

The results suggest that CBD-PLC significantly enhances the permeability of CBD compared to its pure form. This enhancement can be attributed to several factors; the higher P_app_ values for CBD-PLC indicate that it facilitates better transcellular transport across the intestinal epithelial cells. This aligns with findings supporting that PLC can improve drug permeability by altering cell membrane fluidity and utilizing endocytic pathways [24]. The increased recovery percentage for CBD-PLC suggests that a larger fraction of the administered dose is absorbed and transported across the intestinal barrier. This is likely due to the complex’s ability to bypass P-glycoprotein efflux mechanisms and be internalised via endocytosis [63].

**Figure 16:**
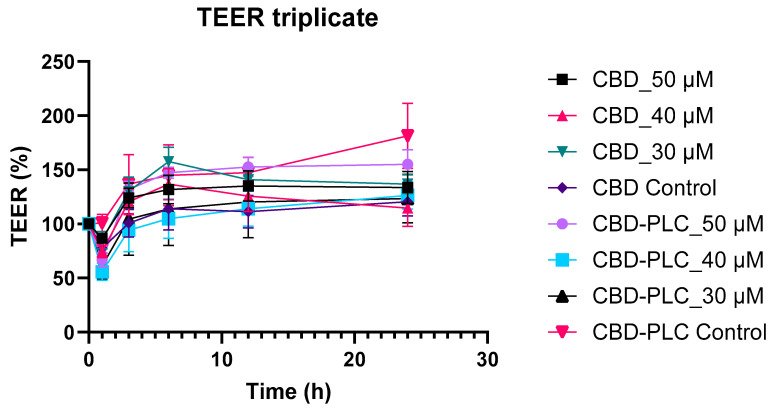
Transepithelial electrical resistance (TEER) readings for CBD, the control, and CBD-PLC, with *n* = 3 ± SD. The x-axis and y-axis represent time (h) and TEER (%), respectively.

The integrity of the Caco-2 monolayer was assessed by measuring TEER across the monolayer using an EVOM2 (World Precision Instruments, Sarasota, FL, USA). TEER was expressed as a percentage of resistance, normalized to the initial value (Figure 16). Analysis of the data reveals a reduction in TEER measurements for all samples at 1 h, including the CBD control. This decrease may be attributed to immediate responses to the media, CBD, or the formulation, potentially involving alterations in tight junctions or cell membrane perturbations. Notably, it has been demonstrated that certain compounds can precipitate a swift decline in TEER due to their impact on cell membranes and cytoskeletal structures, thereby temporarily compromising the integrity of tight junctions [64].

Subsequently, an increase in TEER values was observed for samples treated with CBD alone and the formulation. This recovery and subsequent rise in TEER may be attributed to cellular adaptation or repair mechanisms that restore the integrity of tight junctions over time. Prior studies have indicated that following the initial disruptive impact of specific treatments, cellular mechanisms may enhance tight junction assembly or alter the expression of junctional proteins, thereby strengthening barrier function, as reflected in increased TEER measurements [65].

Indeed, previous investigations have shown that CBD can protect the integrity of the Caco-2 monolayer [66]. However, after 6 h, a decline in TEER values was noted at the lowest concentrations of CBD (40 µM and 30 µM), whereas at 50 µM this effect was not observed. Conversely, in the formulation, TEER readings continued to increase throughout the duration of the study (24 h), suggesting that the formulation may confer superior protective effects compared to CBD alone over extended periods.

This pattern suggests a transient effect of the treatment on cell monolayer permeability, characterised by an initial disruption followed by a recovery or even an enhancement in barrier properties. Such dynamics are common in studies involving permeation enhancers or other compounds that interact with cellular membranes and junctional complexes. In conclusion, the formulation did not compromise the integrity of the monolayer.

## 3. Materials and Methods

### 3.1. Materials/Chemical and Reagents

CBD crystals in powder form were kindly provided by Green Dispensary Compounding (Adelaide, Australia). HPLC-grade methanol and acetonitrile were obtained from EMD Millipore^®^ (Billerica, MA, USA). A Sartorius ultra-pure water system was utilised in all studies (Goettingen, Lower Saxony, Germany). Ethanol was purchased from Thermo Fisher Scientific (Melbourne, Australia). Lipoid E80 (80–85% egg phosphatidylcholine (PC)), 7–9.5% phosphatidylethanolamine (PE), 3% lysoPC, 0.5% lysoPE, 2–3% sphingomyelin, 2% water, 0.2% ethanol, and iodine (value 65–69) were obtained from Lipoid GmbH (Ludwigshafen am Rhein, Rheinland-Pfalz, Germany). PC-98T and PL-100M (68% phosphatidylcholine and 20% phosphatidylethanolamine) were obtained from YST PHARMA CO. Ltd. (Goka-machi, Ibaraki, Japan). L-α-Phosphatidylcholine from dried egg yolk, type X-E, ≥40% (enzymatic), L-α-Phosphatidylcholine from soybean, formic acid, Dulbecco’s Modified Eagle Medium (DMEM), Hanks Balanced Salt Solution (HBSS), Dulbecco’s Phosphate-Buffered Saline (DPBS), Fetal Bovine Serum (FBS), l-glutamine, penicillin/streptomycin (10,000 U/mL), trypan blue solution, acidic isopropanol, D-chloroform, and Corning^®^ Transwell^®^ polyester membrane cell culture inserts (12 mm Transwell with 0.4 μm pore polyester membrane, TC-treated, sterile) were obtained from Merck Pty Ltd. (Sydney, Australia). N-Octanol was purchased from ChemSupply (Adelaide, Australia). CBD-D3 was purchased from Novachem Pty Ltd. (Melbourne, Australia). Capsules CONI-SNAP size #00 (White Opaque) with acid resistance were obtained from Medisca (Sydney, Australia). We used the Stat-ease 360 software (version: 23.1.3) for the Design of Experiments. The Caco-2 cell line was kindly provided by Anthony Wignall, University of South Australia.

### 3.2. Quantification of CBD by HPLC Method

The HPLC method was conducted under the conditions previously studied and optimised by Abdella et al. [67]. Sample analysis was carried out by using an isocratic method using a Luna 5 µm C8(2) 100 Å (250 × 4.6 mm) analytical column at 30 °C, which was connected to an HPLC system (Shimadzu Corporation, Kyoto, Japan) consisting of a photodiode array detector (LC-20ADXR), a degasser (DGU-20A3), a system controller (CBM-20A), an autosampler (SIL-20AHT), a pump (LC20AD), and an LC solution Chromopac data processor. The mobile phase was a mixture of acetonitrile and MilliQ water (8:2, *v*/*v*) eluted at a flow rate of 1.0 mL/min. The sample injection volume was 20 µL, and a wavelength of 220 nm was selected for UV detection of CBD’s peak detected at 7.3 min.

### 3.3. Quantification of CBD by LC-MS Method

The quantities of CBD that permeated through the Caco-2 cells were quantified using LC-MS/MS analysis. The Sciex 6500+ Qtrap LC-MS/MS (Shimadzu, Kyoto, Japan) was used in positive mode with electrospray ionization to analyse the samples. A 5 μL injection volume of each sample was loaded onto a Phenomenex Kinetex C18 analytical column, 100 Å (100 × 2.4 m, 1.7 μm), with a flow rate of 0.4 mL/min. The column temperature was 40 °C. The mobile phases used for LC separation were MilliQ water containing 0.1% formic acid (A) and methanol containing 0.1% formic acid (B). The gradient elution is summarised as follows: 0.1–6.0 min, 2% B; 6.0–7.0 min, 100% B; 8.0–9.0 min, 2% B. The total analysis required 9 min with a dwell time of 100 ms and the source temperature was 400 °C. The MRM transitions for CBD were as follows:

CBD Quantifying 1: 315.1–193.1;

CBD Quantifying 2: 315.1–259.1;

CBD-D3 (Internal Standard) Quantifying 1: 318.1–196.1;

CBD-D3 (Internal Standard) Quantifying 2: 318.1–262.0.

### 3.4. Preparation of CBD-PLC

CBD-PLC was prepared via the solvent evaporation method. Briefly, a predetermined amount of CBD and the PL was accurately weighed and transferred into a 50 mL Falcon tube. Ethanol (30 mL) was then added, and the mixture was vortexed until a homogeneous solution was obtained. The resulting solution was transferred to a 100 mL round-bottomed flask, ensuring complete transfer by rinsing the Falcon tube twice with additional ethanol (5 mL). The flask was placed under reflux and stirred at 40 °C for 30 min.

Following the reaction, the organic solvent was removed using rotary evaporation (Rotavapor R-210, BUCHI Labortechnik AG, Flawil, Switzerland) under reduced pressure, yielding a solid product. The obtained material was further dried under vacuum at room temperature overnight to remove residual solvent. Finally, the dried product was stored at 4 °C in a sealed container until further analysis.

### 3.5. Preliminary Study for Selection of PL

A preliminary investigation was conducted to compare the effects of four different PLs—Lipoid E 80, PC-98T, PL-100M, and L-alpha-phosphatidylcholine from dried egg—when combined with CBD at four different ratios (1:1, 1:5, 1:10, and 1:20). These combinations were then characterised based on their solubility (as outlined in Section 2.7), particle size, and PDI in PBS at pH 6.8.

### 3.6. DLS

Approximately 10 mg of CBD-PLC was dissolved in 2 mL of RO water. The solution was completely dissolved by vortexing and then filtered through a 0.2 μm filter. Subsequently, a 1:10 dilution was made with RO water and allowed to stabilise for a few min away from light before the particle size, PDI, and zeta potential were measured.

### 3.7. CBD Lipophilicity Evaluation (N-Octanol/Water Partition Coefficient)

The solubility profile of CBD-PLC was investigated by adding 100 ± 10 mg of the complex into sealed Falcon tubes containing 5 mL of PBS (pH 6.8), MilliQ water, or n-octanol [68]. The tubes were subjected to gentle agitation on an orbital shaker at room temperature (23 ± 0.5 °C) for a 24 h duration away from light, followed by centrifugation at 3000 rpm for 10 min. The supernatant was subsequently filtered (0.45 μm) and diluted with methanol (1:1) before analysis via HPLC. The *P_o/w_* and its logarithmic form (log *P_o/w_*) are important indicators of the solubility and bioavailability properties of the compounds tested. In this study, three samples (CBD, PM, and CBD-PLC) were evaluated for their partitioning behaviour between octanol and water. The formulas used are as follows:

$$P_{o/w}=\frac{\left[\;sample\right]octanol}{\left[sample\right]\;water}$$

$$Log\;P_{o/w}=\log_{10}\left(P_{o/w}\right)$$

### 3.8. Design of Experiments

In this study, we employed the RSM to optimise the ratio of CBD to the selected PL. Our objective was to achieve optimal performance metrics, including enhanced water solubility, reduced particle size, PDI, and optimal zeta potential. We used the Stat-ease 360 software (version: 23.1.3) to implement an L-optimal design with a quadratic model and a randomised subtype, in line with the study’s objectives and the formulation optimisation process. L-optimal designs are commonly used in the RSM to achieve optimal responses with fewer experiments. The quadratic model, a standard in the RSM, elucidates the relationship between the response and independent variables. Randomising the experimental runs helps minimise potential errors and biases. With a total of six experimental runs and no blocks, this design is well suited for a preliminary study exploring the effects of PLs on CBD. We anticipated that this RSM approach would yield reliable results, guiding the development of an effective CBD oral formulation [69,70].

### 3.9. SEM

A Zeiss Merlin FE-SEM was used for the microscopic characterisation of CBD-PLC. The complex and PM were separately suspended in RO water, and a drop was placed on a stub and allowed to dry at room temperature, protected from light. Microscopic views of the samples were observed at magnifications ranging from 100 to 1000×. The morphology of the CBD-PLC samples and the PM containing CBD and the PL in the same ratio optimised by DoE (0.6378:20) was captured using the secondary electron detector with a low voltage of 1–2 kV. This microscope features a unique charge compensation system that allows for high-resolution imaging of non-conductive samples by sweeping away accumulated electrons on the sample surface with a fine jet of nitrogen.

### 3.10. EE

Approximately 10 mg of CBD-PLC was mixed with 1 mL of acetonitrile in an Eppendorf tube. The mixture was then vortexed for 1 h at low speed using a multi-tube vortex mixer (Melbourne, Australia) [19,70,71]. The samples were then centrifuged at 16,100 rcf for 20 min, resulting in a clear supernatant. This supernatant was filtered through a 0.45 μm syringe filter (PVDF). The results were calculated using the equations described below:

$$EE\;\left(\%\right)=\frac{Total\;amount\;of\;CBD\;added-Free\;“unentrapped\;CBD”}{Total\;amount\;of\;CBD\;added}\times 100$$

### 3.11. UV Spectra

A total of 5 mg of CBD and an equivalent amount of CBD-PLC, the PL, and the PM were dissolved in methanol and scanned by using a UV spectrometer over the wavenumber range of 200–800 nm.

### 3.12. DSC

DSC was performed using a DSC250 (TA Instruments, New Castle, DE, USA) to evaluate the onset temperature, melting point, width of melting events (WME), enthalpy, and crystallinity index (CI) of the pure drug, PM, PL, and CBD-PLC. Approximately 3 mg of each sample was sealed in an aluminium pan and analysed from 25 to 200 °C at a ramp rate of 10 °C/min. Nitrogen was used as a purge gas at a flow rate of 50 mL/min. The WME and CI were calculated using the following equations:

WME(°C)=melting point−onset temperature

(1) CI(%)=(ΔHBM/ΔHSL)×100

### 3.13. FTIR Spectroscopy

The spectra of pure CBD, the PM, the PL, and CBD-PLC were obtained using an Infrared Spectrophotometer Tensor 27 with an attenuated total reflectance module Specac Golden Gate (ATR-FTIR, Bruker, Ettlingen, Germany). A total of 64 scans were performed in a wavenumber range from 4000 cm^−1^ to 400 cm^−1^ at room temperature. The equipment’s diamond window was used for background correction [72].

#### PCA of FTIR Spectra

PCA was conducted on the FTIR spectra for the whole wavenumber range of 400–4000 cm^−1^ using OriginPro 2025 version 10.2.0.188. Adhering to the Kaiser criterion, only principal components with an eigenvalue ≥ 1 were extracted (*n* = 2), yielding a total explained variance of 95% [45].

### 3.14. H-NMR

All ^1^H NMR measurements were obtained using a Bruker Avance III HD spectrometer (Bruker Corporation, Faellanden, Switzerland) at 300 MHz and were analysed using a Bruker Topspin 3.2 program. Chemical shifts are reported in parts per million (ppm) and are referenced to ^1^H signals of residual nondeuterated solvent. For sample preparation, briefly, 5 mg of each sample (pure CBD, PM, PL, and CBD-PLC) was dissolved in 0.5 mL deuterated chloroform, and a total of 16 scans were obtained for each sample [73].

### 3.15. In Vitro Drug Dissolution Study

The dissolution assays were conducted using the paddle methodology (Method II) as specified in the United States Pharmacopoeia (USP 35). The procedure involved placing 90 mL of MilliQ water in a small vessel, which was then submerged in a water bath maintained at a constant temperature of 37 ± 0.5 °C. The paddle speed was set at 100 rpm [74].

Approximately 900 mg of CBD-PLC (the equivalent of 30 mg of CBD), alongside equivalent quantities of pure CBD and a PM of CBD and PL, was encapsulated in acid-resistant size #00 capsules. These capsules were employed as a dosage form, allowing for accurate quantification of the weighed amount of CBD and subsequent calculation of the expected percentage recovery. The capsules were then carefully positioned on the surface of the dissolution medium within each vessel [70].

Samples were collected at predetermined time intervals (0.3, 0.6, 1.0, 1.3, 1.6, 2.0, 3.0, and 6.0 h). To ensure a consistent volume throughout the experiment, fresh dissolution media were added after each sampling. The collected samples were subsequently filtered through a 0.2 μm filter and diluted in a 1:1 ratio with methanol, and 20 μL of the diluted sample was injected into the HPLC system for further analysis.

### 3.16. Stability Study

For the stability study, samples were meticulously incubated within a specialised stability chamber, adhering to the stringent International Council for Harmonisation (ICH) guidelines [75]. HPLC was employed to quantify the drug content. The CBD-PLC samples were placed into small glass vials and subjected to three distinct conditions over a 12-month period:

1. Intermediate conditions at 25 °C with 60% relative humidity, devoid of light exposure;
2. Accelerated conditions at 40 °C with 75% relative humidity;
3. Long-term storage conditions at 4 °C, ensuring optimal preservation.

### 3.17. Intestinal Cell Monolayer Preparation and Treatment

#### 3.17.1. Assessment of Cell Viability in Caco-2 Cells

Caco-2 cells were cultured in DMEM, enriched with 10% (*v*/*v*) Fetal Bovine Serum (FBS), 1% (*v*/*v*) L-glutamine, and 1% (*v*/*v*) penicillin/streptomycin. The cells were incubated at 37 °C in a 5% CO_2_ humidified environment (Sanyo CO_2_ incubator) [66,76].

The viability of these cells was evaluated using the MTT assay (3-(4,5-dimethyl-thiazole-2-yl)-2,5-diphenyltetrazolium bromide, Sigma Aldrich, Melbourne, Australia). In summary, 3.5 × 10^3^ Caco-2 cells were plated in 96-well plates and exposed to varying concentrations of CBD (20–50 µM) for periods of 24, 48, and 72 h. Post-treatment, cells were washed with PBS, followed by the addition of MTT solution (5 mg/mL) diluted in DMEM. After a 3 h incubation period at 37 °C, DMSO was added (150 µL/well) and the absorbance was quantified at 570 nm using a VICTOR™ X3 Multilabel Plate Reader (Perkin Elmer, Waltham, MA, USA) [66,76].

#### 3.17.2. Permeation Study and TEER Assay

Caco-2 cells were seeded on Transwell™ polyester membrane cell culture inserts (transparent PET membrane: 1.0 cm^2^ growth surface area, 0.4 μm pore size; BD Falcon™) in 12-well plates from Merck Pty Ltd. (Sydney, Australia). Cell cultures were maintained at 37 °C, 95% relative humidity and 5% CO_2_. The culture medium was changed every second day. Cells were seeded at a density of 3 × 10^5^ and cultivated for a minimum of 21 days to reach confluency and ensure monolayer differentiation. The integrity of the Caco-2 monolayer was established by measuring TEER across the monolayer using an EVOM2 (World Precision Instruments, Sarasota, FL, USA). The experiment was initiated via the addition of CBD-PLC and CBD (10–50 µM) diluted in PBS in the apical chamber. At predetermined time intervals, 1–3–6–24 h, transport buffer was removed from the apical and basal chamber and immediately replaced with fresh pre-warmed media. Collected aliquots were appropriately diluted with methanol prior to LC-MS analysis, as described previously. All experiments were performed in triplicate, and the data are presented as the mean ± standard error [66,76]. P_app_ and % recovery were calculated using the below equations:

$$Papp=\frac{d_{Q}}{d_{T}}\times \frac{1}{A\times C_{0}}$$

$$\%\;Recovery=100\times \frac{\left[\left(V_{r}\times C_{r}\right)+\left(V_{d}\times C_{d}\right)\right]}{\left(V_{d}\times C_{0}\right)}$$

where:

*d_Q_/d_T_*: the permeability rate.
*A*: the surface area of the cell monolayer.
*C*_0_: the initial concentration in the donor compartment.
*V_r_*: the solution volume in the receiver chamber.
*V_d_*: the volume in the donor chambers.
*C_d_* and *C_r_*: the final concentrations of transport compound in donor and receiver chambers, respectively.

## 4. Conclusions

In this study, a CBD–phospholipid complex (CBD-PLC) was successfully developed using the solvent evaporation method, optimizing the drug-to-phospholipid ratio through a Design of Experiments approach. The complex was comprehensively characterized using FTIR, DSC, and SEM, confirming that encapsulation not only occurs at a physical level but also involves strong chemical interactions. These interactions contributed to enhanced formulation stability, as demonstrated by a 12-month stability study conducted at 4 °C, 25 °C, and 40 °C.

Beyond developing the phospholipid complex, four commonly used phospholipids were systematically screened to identify the most suitable candidate, and a clear, standardized methodology was provided for incorporating phospholipid-based strategies to improve the solubility and permeability of other lipophilic and unstable drugs.

Moreover, the critical role of dissolution media used in in vitro assessments was highlighted. The findings of the present study demonstrate a six-fold increase in solubility and dissolution, with consistent results across both tests. Furthermore, the developed complex enables sustained drug release, which could offer therapeutic advantages by maintaining plasma levels over an extended period.

The significantly enhanced permeability and higher recovery rates observed in the intestinal cell monolayer study indicate the potential of CBD-PLC to improve oral bioavailability. By promoting superior drug absorption in the small intestine, this formulation has the potential to increase the fraction of CBD reaching systemic circulation, thereby enhancing its therapeutic efficacy. Importantly, the cytotoxicity assessment confirmed the complex’s safety.

In conclusion, this study presents a novel and effective phospholipid complexation approach to potentially overcome CBD’s poor absorption, offering a promising strategy for optimizing the oral delivery of lipophilic drugs.

## Figures and Tables

**Table 1:** Intra-day accuracy of developed liquid chromatography–mass spectrometry (LC-MS) method.

Concentration (ng/mL)	Recovery (%)	
	Average	SD
5	99.07	0.62 ^1^
200	101.34	1.46 ^1^
1000	99.48	0.68 ^1^

^1^ *n* = 6.

**Table 2:** The precision of the developed LC-MS method.

Concentration (ng/mL)	CBD Peak Area						Average	RSD (%)
	1	2	3	4	5	6		
5	524,800.0	530,500.0	526,700.0	526,900.0	534,300.0	526,000.0	528,200.0	0.67 ^1^
200	21,180,000.0	20,950,000.0	20,750,000.0	20,380,000.0	2,1070,000.0	21,300,000.0	20,938,333.3	1.59 ^1^
1000	102,700,000.0	103,000,000.0	101,700,000.0	102,800,000.0	104,000,000.0	102,100,000.0	102,716,666.6	0.77 ^1^

^1^ *n* = 6.

**Table 3:** Comparative log P_o/w_ in buffer solution (pH 6.8) using different PLs at varying ratios (*w*/*w*) for formulation screen.

No.	Sample	Ratio	Log P_o/w_
1	Lipe80	1:1	5.0
2		1:5	4.9
3		1:10	4.8
4		1:20	4.6
5	PC-98T	1:1	5.0
6		1:5	4.9
7		1:10	4.8
8		1:20	4.7
9	PL100M	1:1	5.0
10		1:5	4.9
11		1:10	4.0
12		1:20	3.7
13	L-α-Phosphatidylcholine from dried egg yolk	1:1	4.9
14		1:5	4.9
15		1:10	4.8
16		1:20	4.6

**Table 4:** Evaluation of pH, particle size, and polydispersity index (PDI) of various PLs for optimal selection.

**Sample**	**Ratio**	**pH**	**Particle Size (nm)**	**PDI**
Pure CBD	-	-	295.6	0.4
PL-100M	1:10	7.4	432.2	0.4
Lipe80	1:20	6.8	459.4	0.5
Lipe80		7.4	514.5	0.6
PC-98T		6.8	150.3	0.4
PC-98T		7.4	172.6	0.4
L-α-Phosphatidylcholine from dried egg yolk		6.8	215.9	0.3
L-α-Phosphatidylcholine from dried egg yolk		7.4	600.5	0.4

**Table 5:** Design of Experiments (DoE) for determining optimal PL-to-CBD ratio.

Run	CBD	PC-98T	Particle Size	PDI	Log P_o/w_
1	0.5	1	154.76	0.405	5.0
2	1	1	97.83	0.289	5.0
3	1	20	150.33	0.36	4.7
4	0.5	20	85.31	0.402	4.4
5	0.75	20	122.73	0.478	4.5
6	0.75	1	107.8	0.316	5.0

**Table 6:** Confirmation results from DoE for optimal PL-to-CBD ratio.

Analysis	Predicted Mean	Observed	SD	SE Pred	95% PI Low	Data Mean	95% PI High
Particle size (nm)	110.13	145.16	11.05	12.95	54.39	145	165.875
PDI	0.425	0.266	0.052	0.061	0.230	0.266	0.619824

**Table 7:** Comparative analysis of CBD-PLC solubility in different media for dissolution test selection (*n* = 3).

Media	Water Used for the Solution Preparation	CBD Solubility (mg/mL)
MilliQ water	MilliQ water	228.33 ± 0.23
RO water (pH 6.8)	RO water	91.37 ± 0.09
HCl solution (pH 1.2)	MilliQ water	199.36 ± 0.20
	RO water	193.80 ± 0.19
PBS solution (pH 1.2)	MilliQ water	153.69 ± 0.15
	RO water	152.42 ± 0.15
PBS solution (pH 6.8)	MilliQ water	66.58 ± 0.07
	RO water	44.99 ± 0.04
N-octanol	---	310.25 ± 0.31

**Table 8:** Stability study results: drug content and physicochemical properties of CBD-PLC after 6 and 12 months under different temperature conditions.

Sample	Particle Size (nm)	PDI	Zeta Potential (mV)	CBD Concentration (µg/mL)	Recovery % (Expressed as a Percentage of the Day 0 Content)
CBD-PLC Day 0	194.33 ± 16.22	0.277 ± 0.02	−32.57 ± 1.28	299.76 ± 2.80	100
CBD-PLC 12 months at 4 °C	221.27 ± 0.74	0.279 ± 0.01	−46.3 ± 0.43	327.42 ± 18.59	112.16
CBD-PLC 12 months at 25 °C	230.47 ± 0.52	0.278 ± 0.01	−39.1 ± 0.92	298.21 ± 8.85	102.15
CBD-PLC 6 months at 40 °C	192.96 ± 1.68	0.259 ± 0.01	−45.4 ± 0.08	255.04 ± 7.07	86.75

**Table 9:** Apparent permeability coefficient (P_app_, cm/s) and recovery percentage (%) of drug from basolateral side after transport at 6 h.

Sample	Concentration (µM)	P_app_ [AP > BL]	Recovery %
CBD-PLC	30	1.96 × 10^−5^	7.50 ± 0.74
CBD-PLC	40	9.87 × 10^−6^	22.8 ± 0.81
CBD-PLC	50	5.32 × 10^−6^	35.3 ± 3.90
Pure CBD	30	1.48 × 10^−5^	1.20 ± 0.30
Pure CBD	40	5.71 × 10^−6^	3.03 ± 0.12
Pure CBD	50	4.22 × 10^−6^	5.18 ± 1.02

## Data Availability

No new data were created.

## Supplementary Materials

The following supporting information can be downloaded at https://www.mdpi.com/article/10.3390/ijms26062647/s1.
