# Supplementary material for: Optimising Cannabidiol Delivery: Improving Water Solubility and Permeability Through Phospholipid Complexation
Source: Int J Mol Sci. 2025 Mar 14;26(6):2647. doi: 10.3390/ijms26062647 (PMC11942006; doi:10.3390/ijms26062647)
Supplement: Supplementary file 1 [file ijms-26-02647-s001.zip › ijms-3489240-supplementary.pdf]

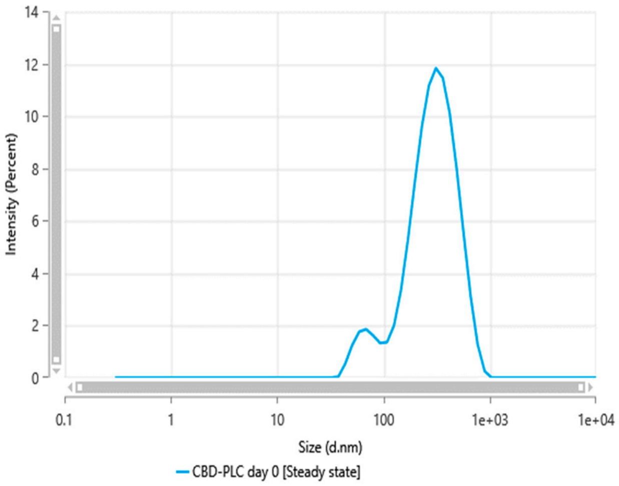

Supplementary Figure S1: Size distribution by intensity of CBD-PLC on day 0, measured in a steady state. The x-axis represents particle size in diameter (d.nm) on a logarithmic scale, while the y-axis displays the intensity percentage. Measurements were conducted using dynamic light scattering (DLS).

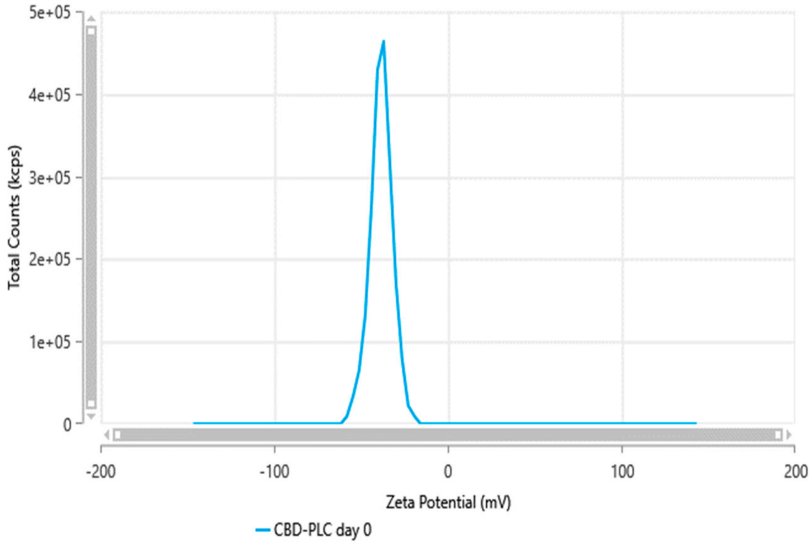

Supplementary Figure S2. Zeta potential distribution of CBD-PLC on day 0, measured in a steady state. The x-axis shows the zeta potential values (mV), while the y-axis represents the total counts (kcps). Measurements were performed using dynamic light scattering (DLS).
